# Sparsentan is superior to losartan in the gddY mouse model of IgA nephropathy

**DOI:** 10.1093/ndt/gfae021

**Published:** 2024-01-25

**Authors:** Hajime Nagasawa, Seiji Ueda, Hitoshi Suzuki, Celia Jenkinson, Yusuke Fukao, Maiko Nakayama, Tomoyuki Otsuka, Teruyuki Okuma, Wilmelenne Clapper, Kai Liu, Mai Nguyen, Radko Komers, Yusuke Suzuki

**Affiliations:** Department of Nephrology, Juntendo University Faculty of Medicine, Tokyo, Japan; Department of Nephrology, Juntendo University Faculty of Medicine, Tokyo, Japan; Division of Kidney Health and Aging, Center for Integrated Kidney Research Advance, Shimane University Faculty of Medicine, Izumo, Japan; Department of Nephrology, Juntendo University Faculty of Medicine, Tokyo, Japan; Department of Nephrology, Juntendo University Urayasu Hospital, Chiba, Japan; Travere Therapeutics, Inc., San Diego, CA, USA; Department of Nephrology, Juntendo University Faculty of Medicine, Tokyo, Japan; Department of Nephrology, Juntendo University Faculty of Medicine, Tokyo, Japan; Department of Nephrology, Juntendo University Faculty of Medicine, Tokyo, Japan; Department of Nephrology, Juntendo University Faculty of Medicine, Tokyo, Japan; Travere Therapeutics, Inc., San Diego, CA, USA; Travere Therapeutics, Inc., San Diego, CA, USA; Travere Therapeutics, Inc., San Diego, CA, USA; Travere Therapeutics, Inc., San Diego, CA, USA; Department of Nephrology, Juntendo University Faculty of Medicine, Tokyo, Japan

**Keywords:** albuminuria, angiotensin II type 1 receptor antagonist, endothelin receptor antagonist, IgA nephropathy, sparsentan

## Abstract

**Background:**

The mechanism leading to the development of immunoglobulin A nephropathy (IgAN) remains to be completely understood. Endothelin-1 (ET-1) as well as angiotensin II (AngII) promote glomerular injury, tubulointerstitial inflammation and fibrosis leading to chronic kidney disease. Sparsentan, a dual endothelin angiotensin receptor antagonist, recently received accelerated approval in the USA for the reduction of proteinuria in adults with IgAN at high risk of disease progression. To elucidate the mechanisms by which sparsentan is efficacious in IgAN, we examined the effect of treatment in gddY mice, a spontaneous IgAN mouse model, versus the monoselective angiotensin II type 1 receptor (AT_1_R) antagonist, losartan, on the development of renal injury at doses resulting in similar blood pressure lowering.

**Methods:**

Four-week-old gddY mice were given control chow, chow containing sparsentan or drinking water containing losartan until 12 or 20 weeks old.

**Results:**

Remarkably, the albumin:creatine ratio (ACR) was attenuated more rapidly and to a greater extent in mice treated with sparsentan than those treated with losartan. The decrease in ACR from baseline after 4 weeks of treatment correlated with beneficial effects of sparsentan on glomerulosclerosis and protection of podocytes and glycocalyx after 16 weeks of treatment across treatment groups; thus, sparsentan treatment delayed development of renal injury to a greater extent than losartan. Expression of mRNA for ET-1, endothelin type A receptor and AT_1_R and proinflammatory genes was upregulated in 12-week-old gddY mice and was prevented by sparsentan and losartan to a comparable extent.

**Conclusions:**

The results of this study, and in light of the results of the phase 3 PROTECT trial, provide a novel perspective and understanding of the mechanisms by which sparsentan has a beneficial renoprotective effect against IgAN compared with AT_1_R antagonism alone.

## Graphical Abstract

**Graphical Abstract:**
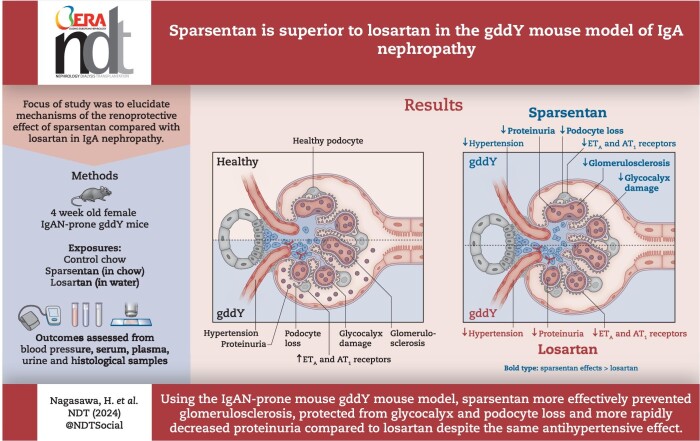

KEY LEARNING POINTS **What was known:**

- The treatment options to prevent progression of renal injury due to immunoglobulin A nephropathy (IgAN) are very limited.
- Sparsentan, a dual antagonist of endothelin angiotensin receptors, has received accelerated approval in the USA for the reduction of proteinuria in adults with IgAN at high risk of disease progression.

**This study adds:**

- Endogenous angiotensin II as well as endothelin systems were up-regulated in the IgAN-prone gddY mouse. Comparison of sparsentan and losartan in the IgAN-prone gddY mouse demonstrated that sparsentan more rapidly decreased ACR, prevented glycocalyx and podocyte damage, and prevented glomerulosclerosis, despite a comparable antihypertensive effect.

**Potential impact:**

- Our observations, taken together with recent results from the PROTECT trial, strongly suggest a novel perspective and understanding of how sparsentan provides a more effective therapeutic option to treat IgAN patients compared with angiotensin II type 1 receptor antagonism alone.

## INTRODUCTION

Immunoglobulin A (IgA) nephropathy (IgAN), first described by Berger and Hinglais [1], is the most common form of primary glomerulonephritis in the world, with the highest prevalence in Asian populations [2]. Up to 40% of the patients progress to end-stage renal disease within 20 years after diagnosis [3]. Diagnosis is based on renal biopsy, which is characterized by mesangial proliferative glomerulonephritis with mesangial deposition of IgA. Abnormalities in the production of IgA1 lead to elevated levels of galactose-deficient IgA1 (Gd-IgA1). IgAN patients have significantly higher levels of circulating IgA1 with galactose-deficient *O-*linked glycans in the hinge region (Gd-IgA1), and this defect represents a risk factor for nephritis. Increased Gd-IgA1 elicits an autoimmune response, resulting in generation of anti-glycan antibodies that recognize N-acetylgalactosamine epitopes on Gd-IgA1 [4]. Mesangial deposits are enriched for IgA1 glycoforms, with some *O*-glycans deficient in galactose [5] (Gd-IgA1), and are bound to Gd-IgA1-specific autoantibodies [6, 7]. It is widely recognized that inflammation signals could be involved in injury of glomerular endothelial cells, podocyte loss and mesangial activation, thus contributing to the development of glomerulosclerosis and interstitial fibrosis in IgAN [8]. It is also well known that excessive activation of renin–angiotensin system (RAS) could be involved in the development of glomerulonephritis [9, 10]; therefore, angiotensin-converting enzyme inhibitors or angiotensin receptor blockers (ARBs) are widely used as first-line treatment for IgAN patients at risk for progressive kidney injury. However, the development of chronic kidney disease (CKD) in IgAN patients is slowed but not prevented by these drugs, highlighting the urgent need for novel therapeutic options.

Endothelin-1 (ET-1) is a vasoactive peptide that is released upon endothelial activation and is an agonist for two G-protein-coupled receptors: endothelin type A receptor (ET_A_R) and endothelin type B receptor (ET_B_R). Activation of ET_A_R results in vasoconstriction, proliferation, inflammation, extracellular matrix production and fibrosis [11–13]. In the healthy kidney, ET_A_R is mainly localized in the smooth muscle cells of the renal vasculature and mesangial cells and podocytes, whereas ET_B_R is abundantly present in the distal nephron as well as in endothelial cells and podocytes [14, 15]. The expression levels of ET-1 and angiotensin II (AngII) have been reported to be increased in IgAN [16, 17] and based on patient biopsies the risk of IgAN progression has been reported to be associated with higher intrarenal expression of ET-1 and AngII [16, 18]. Considering ET-1 actions mediated by the receptor, ET_A_R antagonism is believed to be a novel therapeutic strategy for IgAN patients.

Available clinical evidence suggests antiproteinuric and nephroprotective effects of ET_A_R inhibitors in patients with CKD, including those with IgAN and background RAS blockade [19]. These data support additive effects of ET_A_R and RAS blockade on renal function in patients with or without type 2 diabetes who are at a high risk of developing end-stage kidney disease.

The ongoing phase 3 PROTECT trial (A Randomized, Multicenter, Double-blind, Parallel-group, Active-control Study of the Efficacy and Safety of Sparsentan for the Treatment of Immunoglobulin A Nephropathy) demonstrated superior preservation of kidney function and antiproteinuric effects of sparsentan, a novel, orally active, first-in-class single-molecule dual endothelin angiotensin receptor antagonist, compared with the ARB irbesartan in patients with IgAN [20, 21]. Sparsentan received accelerated approval in the USA for the reduction of proteinuria in adults with IgAN at high risk of disease progression [20]. Although the nephroprotective potential of sparsentan has been demonstrated in the setting of a large clinical trial, the mechanisms of nephroprotection by this drug are still being elucidated.

In this study, we explored mechanisms of nephroprotective effects of the dual antagonism of ET_A_R and angiotensin II type 1 receptor (AT_1_R) using sparsentan compared with standard-of-care treatment losartan [22], a relevant comparator to the ARB moiety of sparsentan, in an experimental model of IgAN.

## MATERIALS AND METHODS

### Mice

The original outbred ddY mouse spontaneously develops IgAN with a variable age of onset. We previously established “grouped ddY” (gddY) mice by the selective mating of ddY mice with the early-onset phenotype for >20 generations [23]. All gddY mice were females and maintained in-house. BALB/c mice (Sankyo Labo Service Corporation Inc., Tokyo, Japan), were used as a healthy control comparison for renal pathology and gene expression studies. All the mice were maintained at the animal facility of Juntendo University (Tokyo, Japan) on normal diet (Oriental Yeast Co., Ltd, Tokyo, Japan) and water *ad libitum* in a specific pathogen-free room. Care was taken in accordance with the NIH Guide for the Care and Use of Laboratory Animals. The experimental protocol of this study was approved by the Ethics Review Committee for animal experimentation of the Juntendo University Faculty of Medicine (permit no. 2 021 268).

### Experimental design

Sparsentan was mixed with normal diet at 900 parts per million (ppm) (SP900) or 1800 ppm (SP1800) (Oriental Yeast Co.), to deliver an estimated equivalent of 180 or 360 mg/kg/day. Doses were selected based on efficacious doses of sparsentan in studies conducted in other murine models and on the daily food consumption of the gddY mice. Losartan (Sigma-Aldrich, St Louis, MO, USA) was provided in drinking water to deliver the equivalent of 10 or 30 mg/kg/day (LS10 or LS30). A pilot study was conducted to assess efficacy of sparsentan, the design is described in the Supplementary Methods.

**Figure 1:**
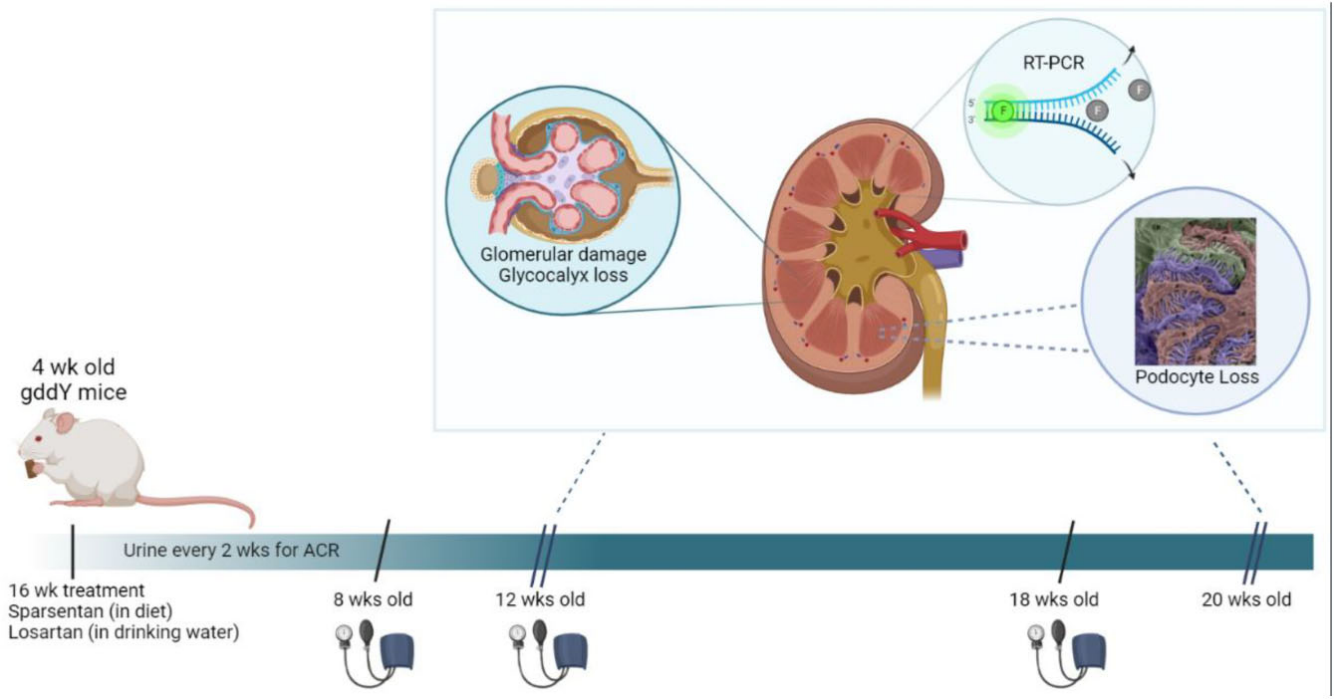
Schematic of study to compare sparsentan and losartan in gddY mice. The 4-week-old gddY mice received sparsentan (900 or 1800 ppm) in their diet or losartan (10 or 30 mg/kg) in drinking water for 8 or 16 weeks, during which urine was collected every 2 weeks for albumin and creatinine determination. Blood pressure was recorded when mice were 8, 12 and 18 weeks old. After 8 weeks of treatment, a subgroup of mice were sacrificed, and the remainder were treated for a total of 16 weeks. Blood was taken for generation of plasma, and kidney samples were removed and processed for assessment of glomerulosclerosis, WT-1 positivity, glycocalyx (20 weeks age only) and gene expression. Created with Biorender.com by W.C. RT-PCR, real-time polymerase chain reaction.

Figure 1 shows a schematic of the main study. gddY mice were treated from 4 weeks of age for 8 or 16 weeks LS10, LS30, SP900 (*n* = 12, 4–12 weeks of age; *n* = 7, 14–20 weeks of age); control gddY mice (gddY C) and SP1800 mice (*n* = 10, 4–12 weeks of age; *n* = 6, 14–20 weeks of age); BALB/c mice (*n* = 10) received regular diet. Body weights (BWs) were measured at the beginning of the study and weekly thereafter. Animals weighed at least 20 g at the start of dosing. Urine was collected to measure ACR at the start of the study and every 2 weeks thereafter at 8 a.m. Urine was stored at −80°C until analysis. Plasma levels of sparsentan in blood samples collected at week 12 and 20 in tubes containing dipotassium ethylenediaminetetraacetic acid (K_2_EDTA) were determined as described in the Supplementary Methods.

### Evaluation of ACR, renal function and blood pressure

Urinary albumin was measured using an albumin enzyme-linked immunosorbent assay (ELISA) (Exocell Inc., Philadelphia, PA, USA) as the sponsor's instructions following spot collection. Urine creatinine levels were also measured in the same samples using creatinine ELISA (Exocell Inc.). ACR was expressed as the urine albumin:creatinine ratio. Serum creatinine levels were measured using an autoanalyzer (Fuji Dry-Chem 5500; Fujifilm, Tokyo, Japan). Systolic blood pressure (sBP) was measured in conscious, restrained mice by tail-cuff plethysmography as previously described [24] (BP-98, Sofron, Tokyo, Japan) at 8, 12 and 18 weeks of age.

### Histologic analyses

Kidney sections (3-μm thick) were fixed in 4% paraformaldehyde and stained with periodic acid–Schiff (PAS) reagent. Histological changes were assessed by light microscopy as previously described [25]. The extent of glomerular damage (glomerular pathological score) was evaluated using a previously described semiquantitative scoring system [26]. In brief, we examined 30 glomeruli per animal (*n* = 5–7 per group) and scored the percentage of glomerulosclerosis by two blinded evaluators (Y.Fukao and M.Nakayama) as follows: 0 = 0%, 1 = 1%–50%, 2 = 50%–99%, 3 = 100%.

Glycocalyx area was determined by immunostaining of 4-μm paraffinized sections. The samples were incubated with FITC-labeled lectin antibodies WGA (F-2101–5; EY Laboratories, Inc., CA, USA) at 4°C overnight and then the positive portion of the glycocalyx in each glomerulus was quantified using ImageJ software (US National Institutes of Health, Bethesda, MD, USA). Ten glomeruli per sample were blindly selected and evaluated by H.Nagasawa, and the values were averaged.

Immunostaining for podocytes was performed on sections incubated with primary anti-Wilms tumor antibodies [rabbit-anti-Wilms tumor protein (WT-1) antibody; ab89901, Abcam, Cambridge, UK] at 4°C overnight and then with secondary antibodies (goat-anti-rabbit-IgG: DAKO) after which the sections were placed in a solution of 3,3′-Diaminobenzidine for staining followed by staining with hematoxylin. The WT-1 positive podocytes in each glomerulus (30 glomeruli per sample) were evaluated by two blinded evaluators (Y.Fukao and M.Nakayama), and the numbers were averaged. Data from kidney tissue from mice at 20 weeks of age where disease had progressed most in the gddY control mice is shown.

### Quantitative PCR assay

RNA from kidney cells was extracted using Trizol solution (Invitrogen, Tokyo, Japan) and purified with RNeasy Mini Kit (74 106; Qiagen, Valencia, CA, USA). Real-time PCR was performed using Taqman Fast Advanced Master Mix (4 444 556: Thermo Fisher Scientific) on a 7500 Fast Real-Time PCR system (Invitrogen). Taqman probes were purchased from Life Technologies (Carlsbad, CA, USA), see Supplementary Methods for details. The quantitative evaluation of mRNA was performed by the ΔΔCT method. Data are shown from mice at 12 weeks of ages when expression changes in ET-1 and AngII system and inflammatory genes between BALB/c and gddY mice were most pronounced.

### Evaluation of serum levels of IgA

Levels of circulating mouse IgA was measured by sandwich ELISA (Bethyl Laboratories, Montgomery, TX, USA).

### Statistical analyses

The JMP (Cary, NC, USA) mixed-model platform was used to create a repeated measures model to assess practical and statistical significance (Supplementary Methods). Tukey HSD multiple comparison procedure was used to determine whether there were significant differences in the mean change in ln (ACR) at treatment week 4 between the treatment levels. The relationship between the change in ln (ACR) at treatment week 4 and the glomerulosclerosis, WT-1 staining and glycocalyx values after 8 (glomerulosclerosis and WT-1 staining only) or 16 weeks of treatment was characterized through exploratory data analysis plots in addition to correlation and regression analysis. Statistical analysis of histological scoring between groups was performed using one-way analysis of variance (ANOVA) and *post hoc* Tukey's test. Analysis of gene expression was conducted using Mann–Whitney U test. Graphs were plotted using GraphPad PRISM software, version 9.0 for Windows (GraphPad Software) or with JMP (SAS Institute, Cary, NC, USA). Data are expressed as mean ± standard error of the mean (SEM) unless otherwise indicated. *P *< .05 was considered significant.

## RESULTS

### Sparsentan lowers ACR more rapidly than losartan in gddY mice

A pilot study conducted in gddY mice treated with sparsentan given in the diet at 900 or 1800 ppm from 4 to 12 weeks of age significantly prevented an increase in ACR compared with gddY control mice (Supplementary data, Fig. S1A). The main study was designed to compare in gddY mice treated from 4 weeks of age for 8 or 16 weeks, at comparable sBP, the ability of sparsentan provided in the diet at 900 or 1800 ppm to attenuate ACR and protect the kidney from injury with that of losartan administered in the drinking water at 10 or 30 mg/kg/day. Plasma levels of sparsentan after 8 weeks of treatment in mice treated with SP900 or SP1800 in the pilot study (Supplementary data, Fig. S1B) were generally comparable to those taken after 8 or 16 weeks of treatment during the main study (Supplementary data, Fig. S1C). The BWs in all groups of mice were not significantly different at 4 weeks of age and BW gains in mice treated with sparsentan and losartan were not statistically different from mice that received normal diet [BW gain at 20 weeks of age, mean ± standard deviation (SD); gddY C: 11.6 ± 0.6 g; SP900: 11.8 ± 0.3 g; SP1800: 11.4 ± 0.8 g; LS10: 11.3 ± 0.4 g; LS30: 10.0 ± 4.4 g].

**Figure 2:**
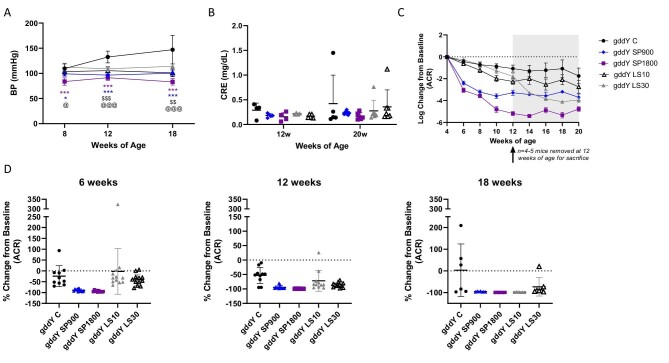
Effects of sparsentan (SP) and losartan (LS) on blood pressure (BP), serum creatinine (CRE) and ACR in gddY mice. (**A**) sBP levels were increased in control gddY mice but attenuated in gddY mice treated with SP or LS. SP (900 ppm) and LS (30 mg/kg) groups had similar antihypertensive effects in 8-, 12- and 18-week-old gddY mice. ***P < .001, **P < .01, *P < .05 for SP900 or SP1800 compared with control gddY, ^@@@^*P *< .001, ^@@^*P *< .01, ^@^*P *< .05 for LS30, and ^$ \$ $$ \$ $$ \$ $^*P *< .001 and ^$ \$ $$ \$ $^*P *< .01 for LS10 compared with control gddY mice following one-way ANOVA and Tukey's multiple comparison (GraphPad PRISM). Data are shown as mean ± SD. (**B**) Serum creatinine remained stable at 12 and 20 weeks of age and was not significantly affected by any treatment during the study. Data are shown as mean ± SD. (**C**) Log change in ACR from baseline over the course of the study. Data are shown as mean ± SD. (**D**) Percentage change in ACR from baseline at 6, 12 and 18 weeks of age (2, 8 and 14 weeks of treatment). Data are shown as mean ± SD for gddY mice C, SP1800 (*n* = 10, 412 weeks old; *n* = 6, 1420 weeks old); SP900, LS10 or LS30 (*n* = 12, 412 weeks old; *n* = 7, 1420 weeks old).

In the main study, the sBP of control gddY mice increased with increasing age. An increase in sBP was significantly prevented by sparsentan and losartan compared with the control gddY mice with SP1800-treated mice, having the greatest magnitude of effect compared with gddY mice at 8, 12 and 18 weeks of age (*P *< .001) (Fig. 2A). There was no significant difference in the BP between gddY SP900 and gddY LS30. Serum creatinine concentrations measured at 12 and 20 weeks of age did not differ between study groups and remained stable throughout the study. ACR was determined bi-weekly during the 16-week study. Most likely because of variability in the onset of disease at 4 weeks of age, baseline ACR was significantly different for the SP900 group compared with control gddY mice or the LS30 group prior to treatment (*P *< .01) (control mean ACR ± SD; control gddY: 8.1 ± 4.1 g/g; SP900: 2.1 ± 1.2 g/g; SP1800: 6.6 ± 3.3 g/g; LS10: 5.2 ± 2.3 g/g; LS30: 7.0 ± 3.9 g/g). ACR in gddY mice treated with SP900, SP1800, LS10 or LS30 decreased relative to that in gddY C, with the most rapid decreases in the sparsentan-treated gddY mice (Fig. 2C and D, and Supplementary data, Fig. S2). After 4 weeks of treatment (8 weeks of age), the decrease in ACR from baseline was greater with either SP900 or SP1800 than with LS10 or LS30. Since there was a difference in baseline ACR prior to treatment, further statistical analysis related to ACR was therefore conducted using the change in ACR from baseline (4 weeks of age; ln transformed). Mixed model analysis showed that all six covariates examined, including treatment, were highly statistically significant (*P *< .0001).

### Sparsentan protected mice from development of glomerulosclerosis, podocyte injury and loss of glomerular glycocalyx after 16 weeks of treatment

Considering similar effects of SP900 and LS30 on sBP structural and molecular statistical analyses are shown only in these groups of mice and gddY C mice and compared with healthy BALB/c mice.

**Figure 3:**
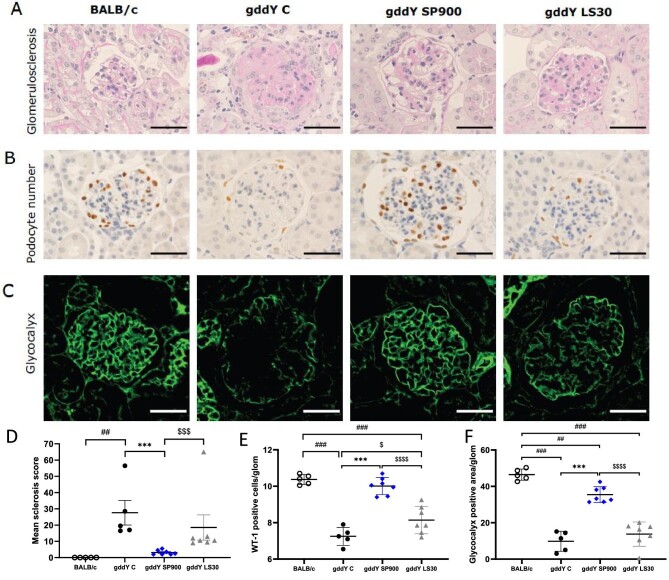
Sparsentan protects from development of glomerulosclerosis, podocyte injury and glomerular glycocalyx to a greater extent than losartan. (**A**) PAS staining, (**B**) staining with anti-WT-1 antibodies, (**C**) immunofluorescence following incubation with FITC-labeled anti-lectin antibodies of representative kidney sections in BALB/c mice or control gddY mice or gddY mice treated with sparsentan 900 ppm in diet or losartan 30 mg/kg/day in drinking water for 16 weeks. Scale bars: 50 μm. (A, **D**) A statistically significant increase in the glomerulosclerosis score was observed in gddY control mice compared with BALB/c mice (*P *< .01). A significant attenuation in glomerulosclerosis was observed in gddY SP900 mice compared with gddY control mice or losartan-treated mice (*P *< .001). (B, **E**) Sparsentan significantly prevented podocyte loss compared with gddY control mice and losartan-treated mice (*P *< .001). Losartan also attenuated podocyte loss in gddY mice but not to the extent of sparsentan, which resulted in gddY mice having a comparable number of podocytes per glomerulus to healthy BALB/c mice. (C, **F**) Sparsentan significantly prevented glycocalyx damage to a greater extent when compared with gddY control mice and losartan-treated mice (*P *< .001). Data are shown as mean ± SD. Statistical analysis in (E) and (F) was performed by one-way ANOVA with *post hoc* analysis using Tukey's multiple comparison test, with the analysis in (E) using ln transformed data. Analysis in (D) for gddY groups was also performed by one-way ANOVA of ln transformed data with *post hoc* analysis using Tukey's multiple comparison test. Comparison of BALB/c and gddY groups in (D) was performed using unpaired *t*-test. ***P < .0001; gddY C vs SP900; ^###^*P *< .0001, ^##^*P *< .01 vs BALB/c; ^$ \$ $$ \$ $$ \$ $$ \$ $^*P *< .0001; ^$ \$ $$ \$ $$ \$ $^*P *< .001, ^$ \$ $^*P *< .05 vs LS30 following one-way ANOVA.

Development of glomerulosclerosis observed in gddY C mice was significantly attenuated by SP900 (*P *< .001), but not LS30. LS30 mice had a significantly higher sclerosis score than SP900 mice (*P *< .001; Fig. 3A and D). No glomerulosclerosis was observed in BALB/c mice as expected. As shown in Fig. 3B and E, podocyte loss observed in gddY C mice was prevented in SP900 and LS30 mice (*P *< .01 and *P *< .05, respectively). However, the number of WT-1-positive cells in SP900 mice was significantly greater than that in LS30 mice (*P *< .001) and was comparable to that in the healthy BALB/c control mice. Sparsentan was also able to prevent the loss of glycocalyx. As shown in Fig. 3C and F, a decrease in the lectin-positive glycocalyx area/glomerulus was observed in gddY C mice compared with BALB/c mice (*P *< .001), which was ameliorated by SP900 (*P *< .001), but not LS30. The preservation of glycocalyx was significantly greater in SP900 than LS30 mice (*P *< .001).

**Figure 4:**
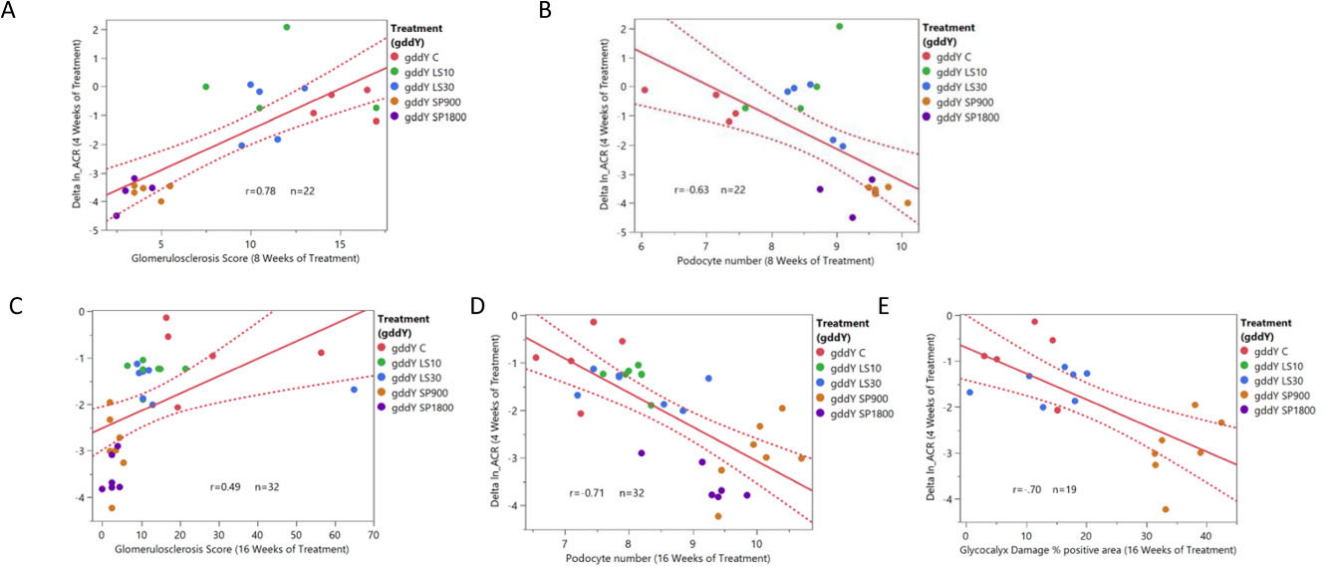
Renal structural changes at weeks 8 and 16 of treatment are correlated with change in ln (ACR) at week 4 of treatment. Relationship between change (delta) in ln (ACR) from week 0 to week 4 of treatment and (**A**) glomerulosclerosis after week 8 of treatment, (**B**) podocyte number (WT-1 immunostaining) after week 8 of treatment, (**C**) glomerulosclerosis after week 16 of treatment. (**D**) podocyte number (WT-1 immunostaining) after 16 weeks of treatment. (**E**) Glycocalyx % lectin positive area after 16 weeks of treatment. *r* = correlation coefficient. Change in ln (ACR) at week 4 is significantly correlated with glomerulosclerosis [*P *< .0001 (A), *P* < .01 (C)], podocyte number [*P *< .01 (B), *P *< .0001 (D)] and glycocalyx damage [*P *< .001 (E)].

Notably, correlation analysis using data from all the treatment groups revealed that the initial change from baseline in ACR at treatment week 4 was statistically correlated with glomerulosclerosis score (*P *< .001) and podocyte number (WT-1, positive cells) (*P *< .01) at treatment week 8 (correlation coefficients of 0.78 and −0.63, respectively), and also at treatment week 16 with glomerulosclerosis score (*P *< .01), podocyte number (*P *< .0001) and glycocalyx damage (% lectin positive area) (*P *< .001), with correlation coefficients of 0.49, −0.71 and −0.70, respectively (Fig. 4).

### Effects of sparsentan or losartan on endogenous endothelin system, AT_1_R and inflammatory and fibrotic genes in gddY mice

**Figure 5:**
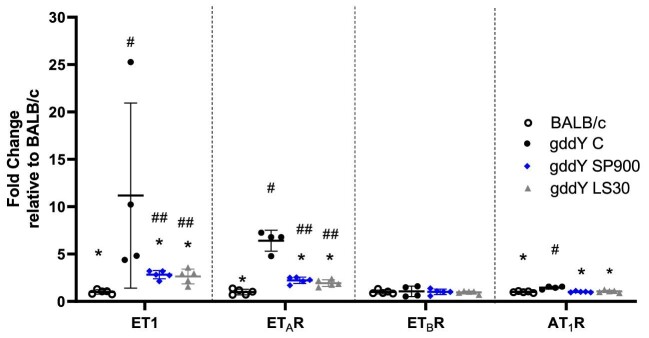
Upregulation of ET-1, ET_A_R and AT_1_R mRNA in 12-week-old gddY mice was prevented by treatment with sparsentan and losartan treatment. Real-time PCR was performed in mRNA isolated from kidney tissue taken from mice at 12 weeks of age. Statistical analysis was performed using Mann–Whitney U test. *P < .05 compared with gddY C, ^#^*P *< .05, ^##^*P *< .01 compared with BALB/c mice. Data are shown as mean fold-change ± SEM relative to expression in BALB/c mice. BALB/c, SP900 and LS30, *n* = 5; gddY C, *n* = 4.

ET-1 and ET_A_R (but not ET_B_R) were strongly upregulated at the mRNA level in the kidneys of gddY C mice when normalized to expression levels in BALB/c mice (*P *< .05) at 12 weeks of age. Increases in expression of these genes was attenuated (*P *< .05) in kidneys of gddY mice treated with SP900 or LS30, although expression of ET-1 and ET_A_R remained significantly elevated compared with the healthy BALB/c mice (*P *< .01; Fig. 5). The increase in AT_1_R mRNA in gddY C mice was significant compared with BALB/c mice (*P *< .05); SP900 and LS30 mice had AT_1_R levels comparable to those of BALB/c mice.

**Figure 6:**
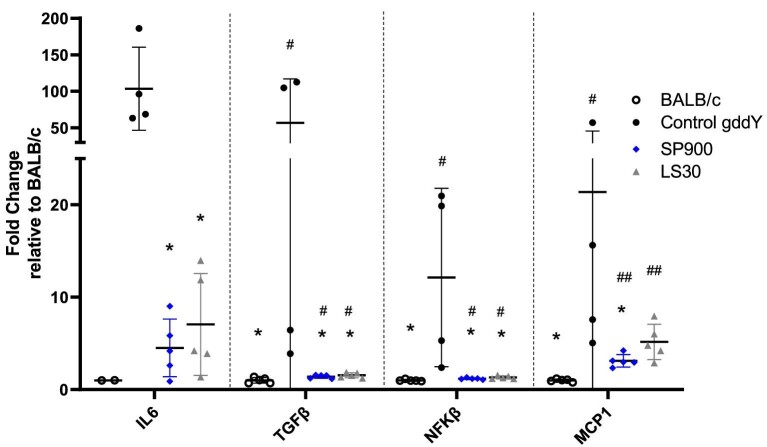
Upregulation of inflammation genes in 12-week-old gddY mice were prevented by sparsentan or losartan. Real-time PCR was performed in mRNA isolated from kidney tissue taken from BALB/c or gddY mice at 12 weeks of age. Statistical analysis was performed using the Mann-Whitney U test. *P < .05 compared with gddY C, ^#^*P *< .05, ^##^*P *< .01 compared with BALB/c mice. Data are shown as mean fold-change ± SEM relative to expression in BALB/c mice. BALB/c, *n* = 5 (except for IL-6, *n* = 2); SP900 and LS30, *n* = 5; gddY C, *n* = 4.

To further examine the mechanisms by which sparsentan could slow down the progression of IgAN in gddY mice, we examined the mRNA expression for inflammatory pathway genes markers including nuclear factor (NF)-κB, interleukin (IL)-6, monocyte chemotactic protein-1 (MCP-1) and fibrotic markers such as transforming growth factor (TGF)-β (Fig. 6).

While mRNA expression of these genes was upregulated to a large extent in control gddY mice compared with BALB/c mice, the increase in expression for IL-6, TGF-β, NF-κB and MCP-1 was attenuated in SP900 mice by 99-, 56-, 11- and 18-fold, respectively, and also in LS30 mice (*P *< .05) by 97-, 55-, 11- and 16-fold, respectively. mRNA expression was significantly lower compared with gddY C mice for all genes in mice treated with SP900 or LS30 (*P *< .05) except for MCP-1 in LS30 mice (Fig. 6).

### Neither sparsentan nor losartan affect glycosylation of IgA in gddY mice

**Figure 7:**
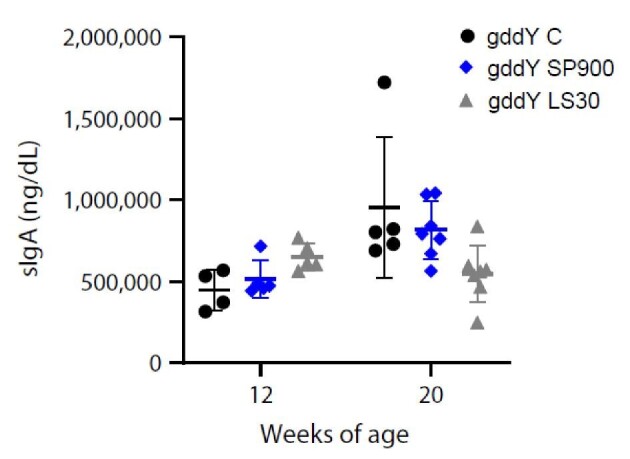
Circulating IgA levels at 12 and 20 weeks of age in gddY mice. Serum levels of IgA were assessed in gddY C, or gddY mice treated with sparsentan or losartan at 12 and 20 weeks of age. Individual animal data are shown with mean and SD.

Figure 7 shows that in gddY C mice serum IgA (sIgA) increased 110% between 12 and 20 weeks of age, but this was not significant. SP900 did not significantly affect sIgA levels at 12 or 20 weeks of age (8 or 16 weeks of treatment). LS30 mice had a small and significant, but likely not meaningful, increase in sIgA of 50% compared with gddY C mice at 12 weeks age (*P *< .05), but levels decreased over time such that LS30 mice had a significantly lower sIgA level compared with gddY C mice at 20 weeks of age (*P *< .05; Fig. 7).

## DISCUSSION

In the present study, treatment with sparsentan attenuated development of ACR and glomerulosclerosis compared with control mice in a model of IgAN. These beneficial effects were associated with prevention of the podocyte and glycocalyx loss and suppression of proinflammatory and profibrotic gene expression. Compared with the ARB losartan used as a representative of SOC, sparsentan exerted a more rapid antiproteinuric effect associated with significantly greater preservation of podocyte number and glycocalyx and protection from glomerulosclerosis. The greater beneficial effects of sparsentan (SP900) compared with losartan (LS30) were observed under conditions of equivalent sBP lowering.

The greater antiproteinuric and potentially nephroprotective effects of dual ET_A_R and AT_1_R inhibition as compared with losartan alone corresponds to clinical evidence accumulated over the past decade both in patients with diabetic nephropathy [19, 27, 28] as well as in patients with nondiabetic glomerular diseases, including those with IgAN. Indeed, in 27 patients with CKD without diabetes, including some with IgAN, who were on optimal RAS inhibition, the selective ET_A_R antagonist, sitaxsentan, reduced 24-h ACR by approximately 30% [29]. Importantly, these current observations are in accord with those in the recently published PROTECT trial which demonstrated substantially greater antiproteinuric effect of sparsentan and long-term preservation of kidney in function compared with the ARB irbesartan in adults with IgAN at high risk of disease progression. This study provides an insight into the mechanisms of action underlying clinical benefit of sparsentan in IgAN and differences from treatment with an ARB alone.

Increased mRNA expression of ET-1 and ET_A_R in gddY mice in this study suggests that the endothelin system is upregulated in experimental IgAN in accordance with clinical findings, including the increased staining for ET-1 in endothelial cells of glomerular and peritubular capillaries reported in patients with IgAN or diabetic nephropathy compared with healthy patients [30]. RAS and inflammation [31] are well known as inducers for ET-1. Our results showed that sparsentan and losartan similarly blocked the upregulation of ET-1 and ET_A_R and mRNA expression of proinflammatory and fibrotic genes, suggesting that AT_1_R signaling could play pivotal roles in regulating the ET-1–ET_A_R axis. The interactions between both the ET-1 and RAS are known to be complex and to include renal cells [22]. ET-1 participates in podocyte and glomerular endothelial dysfunction, inflammation and fibrosis, and promotes mesangial cell proliferation and sclerosis, and its role in the progression of CKD is established [32].

The finding that sparsentan prevented loss of endothelial glycocalyx and podocytes in gddY mice more effectively than losartan suggests a pivotal role of ET-1 and ET_A_R in these cells in IgAN in line with findings in other renal diseases. Heparanase degrades heparan sulfate glycosaminoglycans, a key component of the glycocalyx, and is upregulated in renal epithelial cells in experimental diabetic nephropathy [33]. ET_A_R antagonism increased the endothelial glycocalyx, decreased glomerular heparanase and reduced ACR in mice with diabetic nephropathy [34]. Crosstalk between podocytes and glomerular endothelial cells was found to be key in damage to the endothelial surface layer and subsequent ACR in a manner regulated by ET_A_R in mouse models of focal segmental glomerulosclerosis [35]. In podocytes, ET_A_R activation induces actin cytoskeleton disruption, slit diaphragm dysfunction, basement membrane alterations, apoptosis and inflammation [36]. Persuasive evidence for the role of ET-1 acting via ET_A_R in podocyte pathophysiology was communicated by seminal studies by Buelli *et al*. [37] and Daehn *et al*. [38].

Notably, despite both sparsentan and losartan suppressing the increase in inflammatory gene expression observed in gddY mice, there was no meaningful effect on circulating IgA levels. Underlying molecular mechanisms downstream of the ET_A_ and AT_1_ receptors and their crosstalk involved in the protection from glomerular injury in the gddY model need to be further elucidated. For example, whether sparsentan may differentially affect apoptosis inhibitor of macrophage (AIM), thought to play a role in IgAN progression in the gddY mouse model [25] and in renal fibrosis progression in IgAN patients [39], remains to be explored.

## CONCLUSIONS

This study shows that the ability of sparsentan to protect the gddY mouse model from development and progression of IgAN is beyond effects mediated through a reduction in BP and antagonism of AT_1_R alone and provides mechanistic insight in the currently reported long-term nephroprotective effects of sparsentan in patients with IgAN [21]. Results in the gddY model indicate that the mechanisms that underlie beneficial renal effects and differentiate sparsentan from treatment with an ARB alone include protection from podocyte and glycocalyx loss.

## Supplementary Material

gfae021_Supplemental_Files

## Data Availability

All data are included in the manuscript and/or supporting materials.
