# Supplementary material for: Sparsentan is superior to losartan in the gddY mouse model of IgA nephropathy
Source: Nephrol Dial Transplant. 2024 Jan 25;39(9):1494–503. doi: 10.1093/ndt/gfae021 (PMC11361813; doi:10.1093/ndt/gfae021)
Supplement: gfae021_Supplemental_Files [file gfae021_supplemental_files.zip › 1171 supplementary Materials.docx]

# Supplementary Materials

**Supplementary Methods**

## Determination of SP blood concentrations

Plasma was stored at <−70°C and shipped to Q2 Solutions BioSciences, LLC (Indianapolis, IN). SP-d5 as an internal standard was mixed with 25 µL of mouse plasma (K_2_EDTA), and protein precipitation was performed using solvent (1:1 MeOH:ACN). The supernatant was diluted with water and analyzed by high-performance liquid chromatography with tandem mass spectrometry (AB Sciex 5500). The lower limit of quantification (LLOQ) for analysis of plasma was 1.00 ng/mL

**RT-PCR**

Taqman Probes were as follows: ET-1 (Mm00438656_m1), ET_A_R (Mm01243722_m1), ET_B_R (Mm00432989_m1), AT_1_R (Mm01957722_s1), nuclear factor-κB (NF-κB: Mm00476361_m1), interleukin-6 (IL-6: Mm00446190_m1), monocyte chemotactic protein-1 (MCP-1: Mm00441242_m1) and transforming growth factor-β (TGF-β: Mm01178820_m1). mRNA expression was adjusted relative to the expression of GAPDH (Mm99999915_g1).

**Mixed Model Analysis**

The mixed model establishes the relationship between the change in natural log of ACR from when treatment begins (4 weeks) over time and across the 5 treatments. The fixed effect covariates are the Weeks of age (also the quadratic and cubic terms), the treatment (gddY) and the interaction between these two and all orders of the weeks of age. The animal identification is a random component while the repeated structure uses a first-order autoregressive process to account for correlation within animal effects measured every two weeks. We do note the treatment groups do have different starting point averages for ACR at the beginning of treatment (age 4 weeks). This is accounted for in the mixed model by allowing different intercepts across the 5 treatment levels.

**Supplementary Figures**

**Supplementary Figure S1**. ACR and plasma sparsentan levels in gddY mice treated with SP900 or SP1800 for 8 weeks in the pilot study and plasma sparsentan levels after 8 or 16 weeks of treatment in the main study.

**Supplementary Figure S2**. ACR over the 16-week study in gddY mice.

**Supplementary Figure S3**. Decrease in ACR from baseline with sparsentan 900 ppm is more rapid than that with losartan 30 mg/kg.

**Supplementary** **Figure S1:** ACR and plasma sparsentan levels in gddY mice treated with SP900 or SP1800 for 8 weeks in the pilot study and plasma sparsentan levels after 8 or 16 weeks of treatment in the main study.


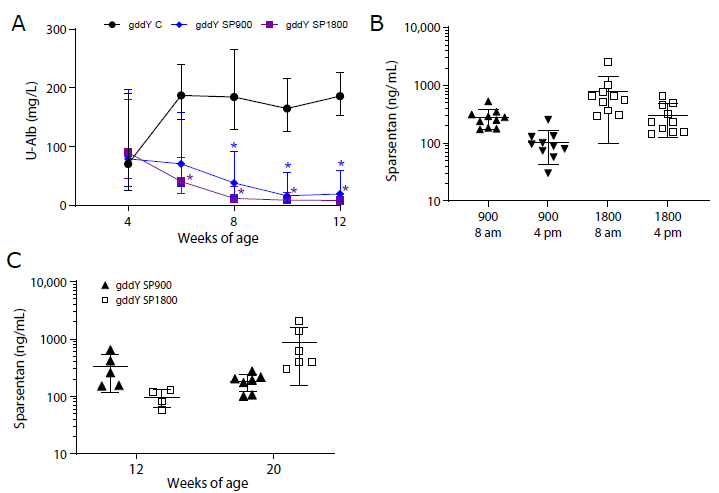


(A) Increase in ACR in pilot study was prevented in mice that received SP900 ppm or SP1800 ppm in diet. **P*<0.05 compared to gddY following two-way ANOVA and Dunnett’s multiple comparison test. (B) Concentration of sparsentan was assessed in plasma collected at 8 AM and 4 PM after 8 weeks of treatment with SP900 or SP1800 ppm in diet in the pilot study. (C) Sparsentan concentration in plasma in 16-week main comparison study with SP900 or SP1800 ppm, plasma taken at 8 AM after 8 weeks of treatment and 12 PM after 16 weeks of treatment. Data are expressed as (A) geometric mean ± SD and (B and C) arithmetic mean ±SD.

**Supplementary** **Figure S2:** ACR over the 16-week study in gddY mice.


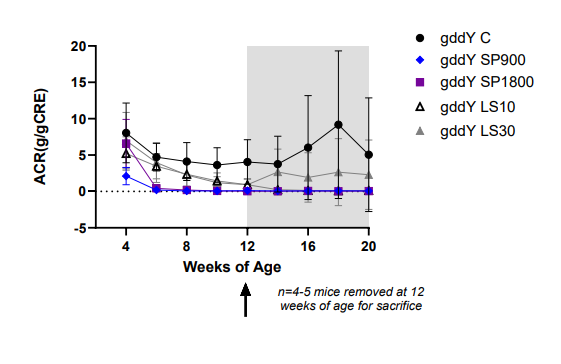


ACR determined in biweekly urine samples in gddY control mice, gddY mice treated with sparsentan in the diet at 900 or 1800 ppm or with losartan in the drinking water at 10 or 30 mg/kg/day. Data shown as mean ±SD. CRE, creatinine.

**Supplementary** **Figure S3:** Decrease in ACR from baseline with sparsentan 900 ppm more rapid than with losartan 30 mg/kg.


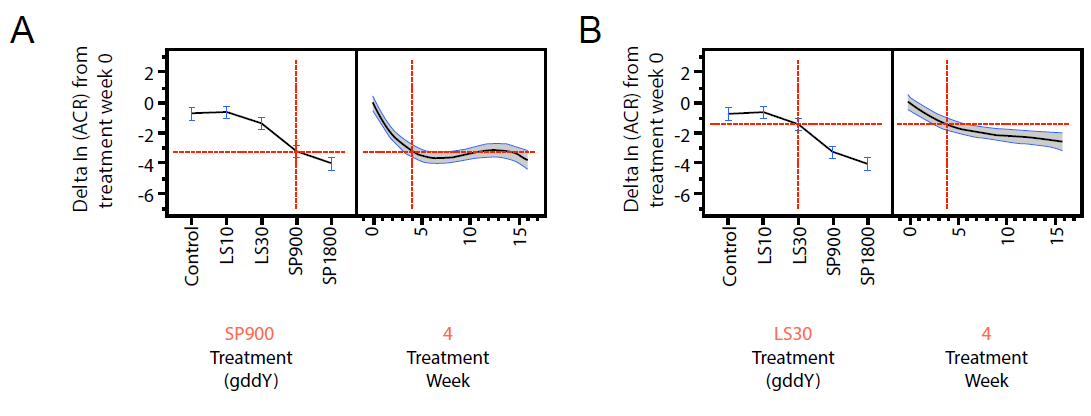


Predicted change in ACR with treatment data following modeling. Mixed model profile prediction for delta ln (ACR) (right-hand sides of Supplementary Fig. S3A compared to Fig. S3B) from treatment week 0 (4 weeks of age) illustrating that the drop in delta ln ACR with sparsentan 900 ppm was more rapid than that with losartan 30 mg/kg. The delta ln (ACR) at treatment week 4 (8 weeks of age) for SP900 treated gddY mice was significantly lower than that in LS30-treated gddY mice indicated by the vertical red dashed line in Supplementary Figure S3A and S3B, respectively.
