# Supplementary figures and images for: Sparsentan is superior to losartan in the gddY mouse model of IgA nephropathy
Source: Nephrol Dial Transplant. 2024 Jan 25;39(9):1494–503. doi: 10.1093/ndt/gfae021 (PMC11361813; doi:10.1093/ndt/gfae021)

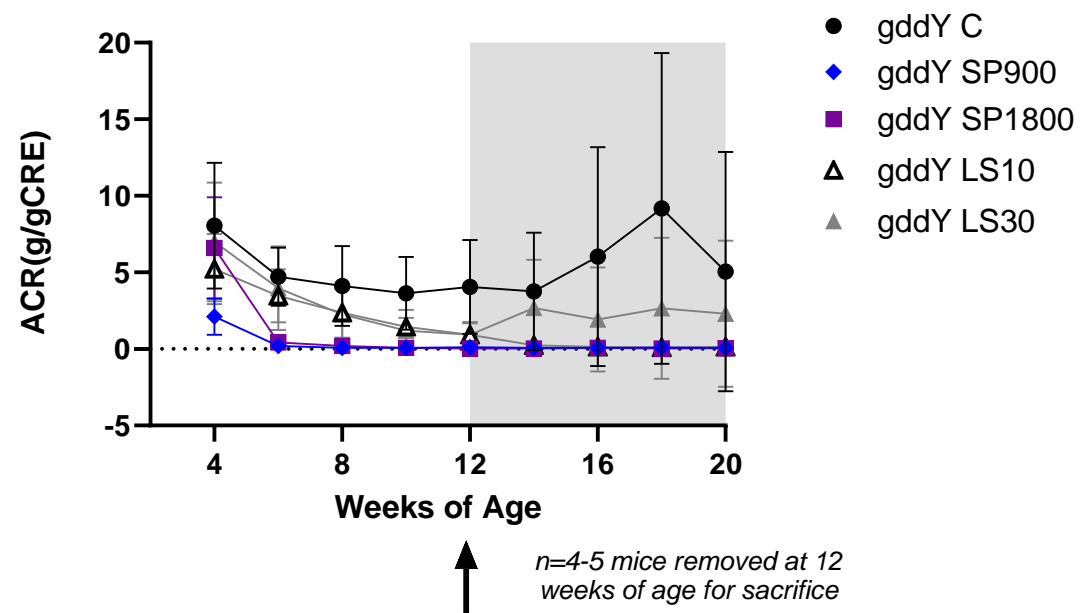

Supplement: gfae021_Supplemental_Files [file gfae021_supplemental_files.zip › SuppFig2_R1.pdf]
